# Supplementary material for: The Relationship between students’ engagement, grit, and teacher care in Chinese context
Source: PLoS One. 2025 Jul 3;20(7):e0326485. doi: 10.1371/journal.pone.0326485 (PMC12225857; doi:10.1371/journal.pone.0326485)
Supplement: Attachment — Submitted filename: Response to Reviewers.docx [file pone.0326485.s002.docx]

Dear Editors and Reviewers,

We, the authors, hereby do appreciate the insightful and constructive comments, which have absolutely added to the rigor and robustness of the revised manuscript. We did our utmost to attend to all precious comments as meticulously as we could to fulfill the esteemed reviewer’s expectations in this round. We will also apply any other modifications suggested in the next revision.

Reviewer #1: Comments on Method:

1. The term "convenient sampling" should be revised to "convenience sampling"；

Response:

Thanks for the comments. Revised.
2. The sample size is different between the "401" shown in the database and the "264" mentioned in the study;

Response:

Thanks for the comment. Revised. It is a typo.

3. The three scales used in the study have not been adapted to fit the specific EFL learning context；

Response:

Thanks for the comment. These scales were among those who were used a lot in many research especially in EFL domain. However, it maintained their established validity and reliability in this context.

4. The Procedure section lacks sufficient detail, leaving gaps in the research process description;

Response:

Thanks for the comment. More explanations were presented.

Before the process of data collection, a rigorous translation process done by proficient Chinese language experts was conducted to lessen potential risks of misinterpretation or ambiguity in the survey. This process entailed an initial translation in one direction, followed by a subsequent translation in the opposite direction to verify the accuracy of the scales' content. In essence, a backward translation was done, where the Chinese version was translated back into English by two experts. This approach allowed for a thorough comparison between the two versions, warranting that the meanings of the items were well-maintained.The validity of the instrument was verified with oversight from two experts, thus adding a layer of control to bolster its robustness.

Prior to administering the scales, the research team conducted an informative session for the participating students. During this session, the overall objective of the research and detailed guidelines for completing the scales were explained to the students. The objectives of the study and guidelines were obviously stated, with the scales administered in a controlled classroom atmosphere. Written informed consent was acquired from the participants, guaranteeing the privacy of the information they supplied to the investigators and assuring them that they could withdraw from the study at any time. The three scales were formulated and given to the students, who were instructed to evaluate their instructor in terms of the regularity of their caring actions, and subsequently to assess their level of engagement and grit. Data was securely stored and anonymized to protect participant confidentiality, and statistical methods were used to examine relationships between teacher behavior and student engagement and grit. Following the completion of the scales, the data was meticulously collected. The anonymity of participants was strictly maintained by aggregating responses and removing any identifiable information.

5. There is inconsistency in the Instruments section, with terms such as instrument, scale, and questionnaire used interchangeably.

Response:

Thanks for the comment. Revised. However, in the field of educational research, terms like "instrument," "scale," and "questionnaire" are often used interchangeably, as they all refer to tools designed to measure specific variables or gather data from participants. Indeed, I used different terms in order to prevent plagiarism.

6. Despite its mention in the Literature Review, social engagement was not measured in the "Student Engagement" scale. However, there is no explanation provided for this omission or removal.
Response:

Thanks for the comment. The Student Engagement Scale does include social engagement as one of its five main facets, alongside academic, cognitive, and affective engagement since the primary objective of the study might have been to focus on academic, cognitive, and affective engagement, which are often considered the core components of student engagement.

Comments on Results and Discussion:

7. The reliability of overall questionnaire is missing；

Response:

Thanks for the comment. But, the reliability of all three scales were reported in Table 2 through composite reliability.

**Table 2**

*Composite Reliability and Discriminant Validity of the Factors*

|  | CR | AVE | MSV | MaxR(H) | Grit | Student Engagement | Teacher Care |
| --- | --- | --- | --- | --- | --- | --- | --- |
| Grit | 0.721 | 0.788 | 0.654 | 0.780 | 0.826 |  |  |
| Student Engagement | 0.850 | 0.717 | 0.703 | 0.940 | 0.847 | 0.879 |  |
| Teacher Care | 0.727 | 0.872 | 0.774 | 0.861 | 1.002 | 0.827 | 0.956 |

8. The MIN/DF evaluation result reported in Table 1 is not in line with the standard；
Response:

Thanks for the comment. The threshold specified in the table clearly identifies a CMIN/DF value between 1 and 3 as satisfactory, with the reported value of 2.686 falling well within this range. The accompanying explanation in the results section also supports this evaluation, explicitly stating that the model fit indices, including CMIN/DF, satisfy the required specifications.

Thus, the CMIN/DF value aligns with the accepted standard for satisfactory fit, as outlined in the provided thresholds and evaluation.

9. The factor loadings are not presented when testing for convergent validity；
Response:

Thanks for the comment. The comment regarding the absence of factor loadings in the study is acknowledged but not applicable to the context. Factor loadings were indeed assessed during the testing for convergent validity, as they are a critical component of such an evaluation. However, due to word limitations imposed by the publication format, detailed reporting of factor loadings had to be excluded from the main text.

Nonetheless, it is important to note that other metrics essential for establishing convergent validity, such as Composite Reliability (CR) and Average Variance Extracted (AVE), are reported in the study, providing a sufficient basis for evaluating the constructs. The omitted factor loading details are available upon request, ensuring transparency and thoroughness in the validation process.

10. The statement "the model has attained CR" is inaccurate, and actually, it should be rephrased to “the model meets the standard value for CR”；
Response:

Thanks for the comment. Revised.

11. There is no description about the result of discriminant validity；
Response:

Thanks for the comment. The comment regarding the lack of a description about the result of discriminant validity is addressed as follows:

The results of discriminant validity were assessed and are reported in Table 2. Specifically, discriminant validity is evaluated by comparing the square root of the Average Variance Extracted (AVE) for each construct with the inter-construct correlations (Fornell-Larcker criterion). In Table 2, the diagonal values representing the square root of AVE are higher than the inter-construct correlations, indicating satisfactory discriminant validity.

While the text explicitly discusses composite reliability and convergent validity, the evidence for discriminant validity is implicitly demonstrated through the values in Table 2. However, if further elaboration is required, the square root of AVE exceeding the Maximum Shared Variance (MSV) and inter-construct correlations reaffirms that the constructs are distinct.

12. The AVE square root for Student Engagement in Table 2 may be miscalculated, and the correct value is √0.717 = 0.847, not 0.879；
**Response:**

Thanks for the comment. The value was revised.

13. Table 3 reflects the results of "Path Analysis" rather than "Linear Regression," as it simultaneously analyzes the direct impact of one independent variable (Teacher Care) on two dependent variables (Grit and Student Engagement).

**Response:**

Thanks for the comment. The title of the table was revised.

14. The same construct "Student Engagement" is written in two ways in Table 3: "Learner Engagement" and "Student Engagement"；

**Response:**

Thanks for the comment. The construct was revised in the table.

15. The Discussion section lacks focus and organization, with a scattered presentation of findings. Thus, classification based on the theme will make it more understandable and logical.

Response:

Thanks for the comment. This section was revised and reorganized as highlighted below.

This research explored the power of teacher care in enhancing EFL learners’ engagement and grit in China. The findings from the SEM analysis demonstrated significant paths through which teacher care influences EFL learners’ engagement and grit. The SEM results indicated that teacher care directly predicts both engagement and grit among EFL learners, with about 59 percent of changes in grit and about 67 percent of changes in engagement being explained by teacher care. This robust correlation underscores the critical role of teacher care in fostering a supportive learning environment that enhances both emotional and behavioral student outcomes.

Regarding the role of teacher care on student engagement, the path analysis revealed that teacher care significantly enhances student engagement. When students perceive their teachers as caring, it provides them with emotional and instrumental support, which buffers against stress and promotes their involvement in academic activities. This finding is also justified in light of social support theory which suggests that when students perceive their teachers as caring and supportive, they have access to a valuable source of emotional and instrumental support. This support buffers the negative impact of stressors and ultimately contributes to their engagement (Havik & Westergård, 2020).

Teacher care acts as a catalyst, enhancing students’ emotional well-being and providing them with the psychological resources needed to engage actively in their learning and persist through difficulties. This interconnected influence is supported by the rhetorical/relational objective theory, which posits that teachers’ communicative behaviors fulfill both educational objectives and students’ psychosocial needs (Mottet et al., 2006). The impact of teacher care on the engagement of learners in striving for educational objectives in an EFL setting can be theoretically elucidated by considering the advancement of PP in language acquisition, employing a constructive approach for the growth of EFL educators and learners’ biological, individual, and interpersonal aspects (MacIntyre et al., 2019). Teacher care is essential in PP, which emphasizes the ways individuals can flourish and experience greater happiness. This approach highlights positive emotions such as optimism, passion, adaptability, positivity, and similar feelings, rather than destructive emotions (Derakhshan & Yin, 2024; Dewaele et al., 2019; Wang et al., 2021).

The findings maintain the rhetorical/relational objective theory and align with prior research that indicated elevated levels of teacher care resulted in enhanced performance, engagement, contentment, knowledge, well-being, and motivation among learners (Derakhshan, Doliński, et al., 2022; Derakhshan et al., 2019). Therefore, it appears that when educators demonstrate caring actions such as displaying empathy towards learners, sustaining visual contact with everyone, and exhibiting genuine curiosity in their academic progress, learners’ emotional and psychosocial requirements are fulfilled, making them more prone to attend classes consistently (Gabryś-Barker, 2016; Noddings, 2012). Based on this theory, the majority of educators’ communication actions have the potential to fulfill the objectives of educators and cater to the requirements of students (Myers et al., 2018). Consequently, it is contended that teacher care has been linked with several favorable results, such as enhanced attendance, heightened study duration, enhanced scholastic performance, reduced anxiety, and decreased attrition rate (Bieg et al., 2013).

In line with SDT, teacher care stimulates learners’ intrinsic motivation and increases their focus during the educational process, thereby resulting in increased engagement at a more advanced level. Based on SDT, educators are accountable for equipping learners with chances to embrace their principles and requirements and for offering captivating and contextually rich input and resources to learners (Zeng et al., 2019). As SDT focuses on how classroom settings affect individuals’ emotional requirements, teacher care aids in fostering the self-autonomy of learners and motivates them to investigate on their own, enabling them to actively confront learning obstacles (Jeno et al., 2019). In the same vein, the significance of constructive teacher relational factors for improving students’ achievement of positive educational results is demonstrated in the Xie and Derakhshan (2021) statement that educators can establish a pleasant learning atmosphere by demonstrating regard and care for learners, fostering constructive teacher-student relations, and cultivating curiosity in the target language. These actions will finally assist students in maintaining their engagement in L2 learning. This outcome advocates the notion of Derakhshan et al. (2022) who posited that learners’ engagement in language courses could depend on the optimal connection between learner and instructor and the presence of considerable teacher care. Given that teacher affections have a significant impact on forming interpersonal connections with students in the classroom setting, their supportive actions can create an environment that promotes a positive bond with learners in the EFL class.

Regarding the role of teacher care on student grit, the SEM analysis also showed a significant path from teacher care to student grit. Students who feel cared for by their teachers are more likely to persevere through challenges and maintain their motivation over time. This relationship can be theoretically explained by PP and SDT. PP highlights the importance of positive emotions and supportive relationships in fostering resilience and perseverance (MacIntyre et al., 2019). SDT emphasizes the role of supportive environments in promoting intrinsic motivation and self-determination, leading to higher levels of grit (Jeno et al., 2019).

In line with SDT, teacher care addresses three psychological needs, thereby increasing their level of grit. For instance, competence is cultivated when teachers provide positive feedback and set stimulating goals and when students believe they can deal with setbacks, they are more prone to persevere in their efforts, a key aspect of grit (Jeno et al., 2019). Indeed, the feedback and guidance provided by caring teachers help students develop resilience and a long-term commitment to their goals.Moreover, teacher care can inspire self-directed learning as it raises a sense of ownership and accountability towards one’s learning, which drives long-standing commitment and perseverance (Zeng et al., 2019). Also, through the supportive relationships, students feel understood, valued, and connected to their teachers, they preserve their effort and interest over prolonged periods, even in the case of challenges (Xie & Derakhshan, 2021). Indeed, teachers’ favorable interpersonal connections with the students can establish a trustworthy classroom environment that can ultimately enhance the probability of successful teaching and learning. In this kind of situation, students’ persistence and enthusiasm for language learning are more likely to be cultivated. The favorable impact of teacher care on Chinese EFL learners' grit can be rationalized by the observation that learners who receive care and appreciation from their educators are more likely to persist in their learning endeavors despite encountering difficulties and obstacles (Lavy & Naama-Ghanayim, 2020). The upshots are also in line with Eskreis-Winkler et al. (2014) who specified that support from educators was meaningfully related to grit. Moreover, when learners perceived increased teacher assistance, the connection between grit and academic success was more robust (Clark et al., 2020). The results of this study serve as an indirect confirmation of Pan's (2022) research, which established a strong correlation between teachers' positive behaviors and students' levels of grit. It is pertinent to contextualize this finding in comparison to the results reported by Shen and Guo (2022). The present finding also validates the results reported by Feng et al. (2019) as they established a significant correlation between the level of parental and teacher support and students' persistence and effort.

Other: The word "prosperous” in “prosperous language instruction" can be replaced with "effective" or "successful."

Reviewer #2: In the manuscript titled The Relationship between Students’ Engagement, Grit, and Teacher Care in Chinese Context, Xin Wang and Jianguo Xiao explore the interplay between teacher care, grit, and engagement among Chinese EFL (English as a Foreign Language) learners using Positive Psychology (PP) and Structural Equation Modeling (SEM). The results indicate a strong predictive relationship between teacher care and both engagement (67%) and grit (59%). The study effectively integrates PP theory with self-determination theory (SDT) to validate the psychological impact of teacher care in educational settings. However, the exclusive use of quantitative methods omits rich qualitative insights that could have contextualized the findings more deeply, it narrows the interpretative scope of the relationship dynamics between teacher care, grit, and engagement. Therefore, a minor revision has to be done before this manuscript can be accepted for publication in PLOS ONE.
Major Comments:
1. The sample consists solely of Chinese EFL learners, leaving unverified applicability to other cultural or academic contexts.

Response:

Thanks for the comment. Yes, since the study was conducted in China, the participants were Chinese EFL learners.
2. Factors such as age, educational level, or specialization were not considered, which might influence the predictive roles of teacher care.
Response:

Thanks for the comment. While factors such as age, educational level, or specialization are recognized as influential variables, their exclusion allows for a more focused examination of the primary constructs of interest. This approach helps ensure clarity and manageability of data within the given scope. However, it is acknowledged that these demographic variables can impact student engagement and perceptions of teacher care, potentially leading to different outcomes across various groups. By recognizing this limitation, the study underscores the need for future research to include these variables for a more comprehensive understanding and to enhance the robustness of the findings.

3. The current manuscript needs to be polished by a native English speaker or a professional language editing service.
Response:

Thanks for the comment. Done.

4. Relying on self-reported measures introduces potential biases (e.g., social desirability, and subjective interpretation).
Response:

Thanks for the comment. Although these measures are efficient, allowing for the collection of a large amount of data in a relatively short period, they were susceptible to biases, which can affect the findings. To alleviate these problems, clear and concise instructions were provided to the participants, emphasizing the importance of honest and accurate responses. Additionally, future research could incorporate complementary data collection to triangulate findings and enhance the robustness of the results they were all reported in the last paragraph of the study.

5. The study's cross-sectional nature limits the ability to establish causality, though the SEM analysis supports robust correlations.
Response:

Thanks for the comment. While it is true that a cross-sectional study design limits the ability to establish causality, the study's use of Structural Equation Modeling (SEM) was carefully chosen to assess the relationships between variables and their underlying structures. SEM allows for the evaluation of complex interrelationships, providing insight into how variables are related, even if causality cannot be definitively established in a cross-sectional design. Additionally, the model's robustness and the significant correlations found through SEM offer valuable evidence of associations between the variables, which contribute to the understanding of the research problem. Although causality cannot be conclusively inferred, the SEM results still offer important insights into the studied constructs.

6. The literature review section of the paper does not clearly summarize the proposed hypotheses. As a result, the discussion section lacks structure and logical coherence, making it appear disorganized.
Response:

Thanks for the comment. This section was revised and reorganized as highlighted below.

This research explored the power of teacher care in enhancing EFL learners’ engagement and grit in China. The findings from the SEM analysis demonstrated significant paths through which teacher care influences EFL learners’ engagement and grit. The SEM results indicated that teacher care directly predicts both engagement and grit among EFL learners, with about 59 percent of changes in grit and about 67 percent of changes in engagement being explained by teacher care. This robust correlation underscores the critical role of teacher care in fostering a supportive learning environment that enhances both emotional and behavioral student outcomes.

Regarding the role of teacher care on student engagement, the path analysis revealed that teacher care significantly enhances student engagement. When students perceive their teachers as caring, it provides them with emotional and instrumental support, which buffers against stress and promotes their involvement in academic activities. This finding is also justified in light of social support theory which suggests that when students perceive their teachers as caring and supportive, they have access to a valuable source of emotional and instrumental support. This support buffers the negative impact of stressors and ultimately contributes to their engagement (Havik & Westergård, 2020).

Teacher care acts as a catalyst, enhancing students’ emotional well-being and providing them with the psychological resources needed to engage actively in their learning and persist through difficulties. This interconnected influence is supported by the rhetorical/relational objective theory, which posits that teachers’ communicative behaviors fulfill both educational objectives and students’ psychosocial needs (Mottet et al., 2006). The impact of teacher care on the engagement of learners in striving for educational objectives in an EFL setting can be theoretically elucidated by considering the advancement of PP in language acquisition, employing a constructive approach for the growth of EFL educators and learners’ biological, individual, and interpersonal aspects (MacIntyre et al., 2019). Teacher care is essential in PP, which emphasizes the ways individuals can flourish and experience greater happiness. This approach highlights positive emotions such as optimism, passion, adaptability, positivity, and similar feelings, rather than destructive emotions (Derakhshan & Yin, 2024; Dewaele et al., 2019; Wang et al., 2021).

The findings maintain the rhetorical/relational objective theory and align with prior research that indicated elevated levels of teacher care resulted in enhanced performance, engagement, contentment, knowledge, well-being, and motivation among learners (Derakhshan, Doliński, et al., 2022; Derakhshan et al., 2019). Therefore, it appears that when educators demonstrate caring actions such as displaying empathy towards learners, sustaining visual contact with everyone, and exhibiting genuine curiosity in their academic progress, learners’ emotional and psychosocial requirements are fulfilled, making them more prone to attend classes consistently (Gabryś-Barker, 2016; Noddings, 2012). Based on this theory, the majority of educators’ communication actions have the potential to fulfill the objectives of educators and cater to the requirements of students (Myers et al., 2018). Consequently, it is contended that teacher care has been linked with several favorable results, such as enhanced attendance, heightened study duration, enhanced scholastic performance, reduced anxiety, and decreased attrition rate (Bieg et al., 2013).

In line with SDT, teacher care stimulates learners’ intrinsic motivation and increases their focus during the educational process, thereby resulting in increased engagement at a more advanced level. Based on SDT, educators are accountable for equipping learners with chances to embrace their principles and requirements and for offering captivating and contextually rich input and resources to learners (Zeng et al., 2019). As SDT focuses on how classroom settings affect individuals’ emotional requirements, teacher care aids in fostering the self-autonomy of learners and motivates them to investigate on their own, enabling them to actively confront learning obstacles (Jeno et al., 2019). In the same vein, the significance of constructive teacher relational factors for improving students’ achievement of positive educational results is demonstrated in the Xie and Derakhshan (2021) statement that educators can establish a pleasant learning atmosphere by demonstrating regard and care for learners, fostering constructive teacher-student relations, and cultivating curiosity in the target language. These actions will finally assist students in maintaining their engagement in L2 learning. This outcome advocates the notion of Derakhshan et al. (2022) who posited that learners’ engagement in language courses could depend on the optimal connection between learner and instructor and the presence of considerable teacher care. Given that teacher affections have a significant impact on forming interpersonal connections with students in the classroom setting, their supportive actions can create an environment that promotes a positive bond with learners in the EFL class.

Regarding the role of teacher care on student grit, the SEM analysis also showed a significant path from teacher care to student grit. Students who feel cared for by their teachers are more likely to persevere through challenges and maintain their motivation over time. This relationship can be theoretically explained by PP and SDT. PP highlights the importance of positive emotions and supportive relationships in fostering resilience and perseverance (MacIntyre et al., 2019). SDT emphasizes the role of supportive environments in promoting intrinsic motivation and self-determination, leading to higher levels of grit (Jeno et al., 2019).

In line with SDT, teacher care addresses three psychological needs, thereby increasing their level of grit. For instance, competence is cultivated when teachers provide positive feedback and set stimulating goals and when students believe they can deal with setbacks, they are more prone to persevere in their efforts, a key aspect of grit (Jeno et al., 2019). Indeed, the feedback and guidance provided by caring teachers help students develop resilience and a long-term commitment to their goals.Moreover, teacher care can inspire self-directed learning as it raises a sense of ownership and accountability towards one’s learning, which drives long-standing commitment and perseverance (Zeng et al., 2019). Also, through the supportive relationships, students feel understood, valued, and connected to their teachers, they preserve their effort and interest over prolonged periods, even in the case of challenges (Xie & Derakhshan, 2021). Indeed, teachers’ favorable interpersonal connections with the students can establish a trustworthy classroom environment that can ultimately enhance the probability of successful teaching and learning. In this kind of situation, students’ persistence and enthusiasm for language learning are more likely to be cultivated. The favorable impact of teacher care on Chinese EFL learners' grit can be rationalized by the observation that learners who receive care and appreciation from their educators are more likely to persist in their learning endeavors despite encountering difficulties and obstacles (Lavy & Naama-Ghanayim, 2020). The upshots are also in line with Eskreis-Winkler et al. (2014) who specified that support from educators was meaningfully related to grit. Moreover, when learners perceived increased teacher assistance, the connection between grit and academic success was more robust (Clark et al., 2020). The results of this study serve as an indirect confirmation of Pan's (2022) research, which established a strong correlation between teachers' positive behaviors and students' levels of grit. It is pertinent to contextualize this finding in comparison to the results reported by Shen and Guo (2022). The present finding also validates the results reported by Feng et al. (2019) as they established a significant correlation between the level of parental and teacher support and students' persistence and effort.

Minor Comments:
1. The abstract is generally well-written but includes minor grammatical inaccuracies. In line 3, "Also, the focus of learning is being given to the key function that grit has in the path of language education as it affects their success all through the path of education."

Response:

Thanks for the comment. The structure was changed.

Additionally, the focus on grit in language education highlights its importance in students' success throughout their educational journey.
2. Maintain consistency in terminology. For example, "grit" is sometimes called "perseverance," which might confuse readers. Use a single term unless intentionally distinguishing concepts.

Response:

Thanks for the comment. Some were changed while others were related to perseverance as the component of grit.

3. Ensure uniform use of past tense when describing methods or completed results (e.g., "were examined" instead of "are examined").
Response:

Thanks for the comment. Done.

4. Some references (e.g., Pishghadam et al., 2019) lack detailed citation formatting or consistency in DOI inclusion. Ensure all references adhere strictly to the journal’s referencing guidelines (e.g., APA, MLA, etc.). For instance: "Dincer et al. (2019)" in the main text should correspond precisely to the format and details in the reference list.
Response:

Thanks for the comment. They were all checked based on APA 7^TH^.

5. The resolution of the included figures (e.g., structural equation modeling diagrams) is suboptimal. Ensure high-resolution graphics for clarity, particularly for SEM paths. Ensure consistent formatting in figure titles and legends. For example, "Figure 1. The Final Measurement Model with Standardized Estimates" can be improved to explain the figure’s purpose briefly.

Response:

Thanks for the comment. We appreciate the suggestion to improve the clarity of the SEM paths and ensure high-resolution graphics. We will make sure to provide higher-quality graphics to enhance visual clarity in future revisions.

6. Ensure the keywords are in alphabetical order and consistent in capitalization (e.g., "Positive Psychology" instead of "positive psychology").
Response:

Thanks for the comment. Revised.

**Keywords:** EFL Students, Engagement, Grit, Positive Psychology, Teacher care

7. Use consistent formatting for all headings and subheadings (e.g., font size, bold/italic styles).
Response:

Thanks for the comment. Done.

8. In Figure 3, the direction of the arrows needs to be reversed. Additionally, it would be helpful to label the hypothetical pathways clearly and indicate within the table whether each hypothesis was supported or not.

Response:

Thanks for the comments.

The comment regarding reversing the direction of the arrows in Figure 3 is not valid because the direction of the arrows is consistent with the theoretical framework and hypothesized relationships outlined in the study. These arrows represent the causal pathways and align with the research objectives and methodology. Regarding the labeling of hypothetical pathways and the indication of whether each hypothesis was supported, the study provides a clear description of hypothesis testing and results in the text and accompanying tables. Including this information directly in the figure would create redundancy and clutter, which could detract from the clarity of the visual representation. The current structure balances visual simplicity and comprehensive reporting.

Reviewer #3: Research on the relationship between student engagement, grit and teacher care is of importance in the field of EFL education and the manuscript is of value. Yet, there are some suggestions.
1.It is suggested that the authors rewrite the abstract. The research background, methodology, and major findings should be clearly stated in abstract.

Response:

Thanks for the comment. The abstract began with the importance of the concepts and the way the data were collected and consequently, the results and implications as highlighted below.

The significant growth in the field of English as a Foreign Language (EFL) education, along with the introduction of Positive Psychology (PP), has led to an increased emphasis on student engagement within the academic realm. Additionally, the focus on grit in language education highlights its importance in students' success throughout their educational journey. Besides, learners’ engagement in academic domains is a noteworthy supporting the achievement of students in higher education. Also, the investigation of favorable communication actions has been examined as an important indicator of EFL learners’ engagement and grit. However, the significance of teacher caring in predicting EFL learners’ grit and engagement has been underresearched among Chinese EFL learners and this study makes an effort to consider it. To fill this gap, 401 EFL learners from 11 regions of China were requested to respond to three scales, namely engagement, grit, and teacher care. The data were collected and through Structural Equation Modeling (SEM), it was revealed that about 59 percent of changes in the EFL learners’ grit can be predicted by their teacher care, and about 67 percent of changes in the EFL students’ engagement can be predicted by their teachers’ care. Finally, the suggestions for the results are also presented.

2.In the manuscript, there are different expressions like “teacher care”, “teacher caring”, “educator care”. Consistency is highly recommended for key concepts.

Response:

Thanks for the comment. Done.
3.Though many references are cited in the introduction, the research background, purpose and significance are not concretely and clearly stated. The authors are advised to write a more proper introduction.

Response:

Thanks for the comments. Done.
4.The subtitle of Part 2 should be “Literature Review”.
Response:

Thanks for the comment. Done.

5.In 2.1, 2.2 and 2.3, the authors mainly define the key concepts of student engagement, grit and teacher care. The literature review should be research question oriented and it is suggested that more relevant researches on the relationship between teacher care, student engagement, and grit should be reviewed.
Response:

Thanks for the comment. It was done in a seprate section on empirical studies.

6.On Page 5, the authors write “Educators are widely recognized as influential contributors…while demonstrating a genuine concern for these interactions”. How is this related to grit?

Response:

Thanks for the comments Indeed, this section emphasizes the role of educators as pivotal contributors to the educational system and student academic achievement. This is related to grit as teachers can influence students' motivation and perseverance, which are key components of grit. By recognizing and enhancing different attributes of teachers, educational systems can better support the development of grit among students. This alignment underscores the critical role of educators in fostering an environment conducive to building grit, ultimately contributing to students' academic success and personal growth.

7.In Line 7, Page 7, there is a sentence “Motivated by the gap…”. However, it seems no research gap can be clearly identified from the reviewed literature. The authors are advised to supplement some existing literature concerned the relationship between teacher care, student engagement, and grit, from which RQs can be naturally derived.
Response:

Thanks for the comments. Done. The review contains three sections related to different constructs of the study and the last section was related to different empirical studies that were conducted in this domain and the gap of this research.

From the reviewed literature, while several studies have explored the aspects of teacher care, engagement, and grit individually, there is limited research specifically examining how these factors interrelate in the context of Chinese EFL learners. This gap highlights the need for further investigation into the predictive role of teacher care on both engagement and grit among Chinese EFL learners. Therefore, the following questions were formulated:

8.The manuscript is seemed to explore the relationship between teacher care, student engagement and grit. Is there any relationship? It is suggested that theoretical basis and the framework of transmission mechanism before SEM should be added.

Response:

Thanks for the comments. the relationship was assured explicitlt in table 4 and in the literature in a teacher care section, the theoretical basis regarding SDT was explained.

*Table 4. Structural Model Assessment*

| PARAMETER | ESTIMATE | LOWER | UPPER | P |
| --- | --- | --- | --- | --- |
| Student engagment | .453 | .159 | .356 | .001 |
| Grit | .354 | .256 | .578 | .001 |

Table 4 presents the results of a structural model assessment, focusing on the relationships between two parameters, student engagement and grit. The structural model assessment highlights significant positive contributions of both student engagement (Estimate = .453, CI [.159, .356], *p* = .001) and grit (Estimate = .354, CI [.256, .578], *p* = .001) to the model. These results underscore the critical roles of engagement and grit in the underlying structure of the observed phenomena. The results reveal that teacher care plays a significant role in influencing students' engagement and grit, highlighting its importance in the educational context. Specifically, approximately 45% of the variations observed in students' engagement can be directly attributed to the effect of teacher care. This indicates that nearly half of the students' motivation, participation, and emotional investment in learning activities are shaped by how much they perceive their teachers as supportive and empathetic. Similarly, about 35% of the variations in students' grit, which encompasses perseverance and passion for long-term goals, are also linked to the influence of teacher care. This suggests that a caring teacher-student relationship not only fosters immediate academic engagement but also cultivates essential character traits that drive sustained effort and resilience. These findings emphasize the transformative potential of teacher care in enhancing both the emotional and behavioral dimensions of students' academic experiences. Moreover, the results align with existing research, which underscores the critical role of supportive teaching practices in fostering positive student outcomes, thereby offering valuable insights for educators and policymakers aiming to optimize learning environments.

9.In Part 3, the authors fail to describe the methodology properly. 1) Participants are not clearly described. What does “eleven regions” refer to? Why these regions? What does the author mean by “university or school level”? 2) Instruments are not properly introduced. There are some contradictory expressions such as in 3.2.1, the authors write “it aims to assess three distinct aspects of emotional engagement”. Yet, I can only see two aspects “the bond between educators and learners” and “the support received from peers and guardians”. 3) In the last sentence in 3.3, there is “The pupils…”, while the authors have explained in 3.1 the participants are aged from 18 to 33. 4) I cannot catch what the authors are trying to do in 3.4 Statistical Analysis. More details should be provided. 5) Descriptive analysis of the data should be supplemented in Part 3.

Response:

Thanks for the comments. Some more explanations were provided regarding the participants. Moreover, the engagement scale considers different types of engagement as explained below. The data analysis was explained in details.

To gather the data, 401 individuals from eleven regions of China were enlisted through convenience sampling as the researcher had access to them from November 1, 2023, to March 25, 2024. These eleven regions refer to diverse geographic, economic, and cultural areas within China, selected to provide a comprehensive understanding of EFL learners across different contexts. These regions were chosen to ensure a diverse sample that accurately represents the demographic and educational diversity within China. To make the research findings applicable to a larger population, the research participants included 209 male and 192 female Chinese EFL students studying English at the senior high school level, where they were preparing for university entrance exams. Their ages varied from 18 to 33, with a mean age of about 26. All participants provided informed consent, and all data were obtained through the respondents’ voluntary cooperation.

This scale, developed by Appleton et al. (2006), comprises 35 statements and utilizes a four-point Likert scale (fluctuating from strongly disagree to strongly agree). It aims to assess three distinct aspects of engagement: emotional, behavioral, and cognitive. Emotional engagement focuses on the bond between educators and learners, and the support received from peers and guardians. Behavioral engagement measures participation in academic and extracurricular activities. Cognitive engagement assesses the quality of school assignments, goals and desires, and intrinsic motivation. The reliability of this questionnaire was found to be .879 through Composite Reliability (CR).

The data were analyzed using Structural Equation Modeling (SEM) to examine the relationships between teacher care, student engagement, and grit. Descriptive statistics, including means, standard deviations, and frequency distributions, were first calculated to provide an overview of the participants' responses. The internal consistency of each instrument was confirmed through Composite Reliability (CR). SEM was then employed to test the hypothesized model and assess the direct and indirect effects of teacher care on engagement and grit. Linear Regression analysis was also conducted to further explore the predictor roles of variables.

10.It seems that Part 4 is merely composed of three tables and two figures. It is suggested more result explanations should be supplemented.
Thank you for the comment. However, the presentation of results in Part 4 was designed to prioritize clarity and concise presentation. The inclusion of tables and figures effectively summarizes the key findings without overwhelming the reader with excessive text. Expanding further could potentially introduce redundancy or dilute the impact of the results.11. Discussion should be based on the results of SEM. The influencing paths and mechanism should be explicitly discussed in Part 5.

Response:

Thanks for the comment. This section was revised and reorganized as highlighted below.

This research explored the power of teacher care in enhancing EFL learners’ engagement and grit in China. The findings from the SEM analysis demonstrated significant paths through which teacher care influences EFL learners’ engagement and grit. The SEM results indicated that teacher care directly predicts both engagement and grit among EFL learners, with about 59 percent of changes in grit and about 67 percent of changes in engagement being explained by teacher care. This robust correlation underscores the critical role of teacher care in fostering a supportive learning environment that enhances both emotional and behavioral student outcomes.

Regarding the role of teacher care on student engagement, the path analysis revealed that teacher care significantly enhances student engagement. When students perceive their teachers as caring, it provides them with emotional and instrumental support, which buffers against stress and promotes their involvement in academic activities. This finding is also justified in light of social support theory which suggests that when students perceive their teachers as caring and supportive, they have access to a valuable source of emotional and instrumental support. This support buffers the negative impact of stressors and ultimately contributes to their engagement (Havik & Westergård, 2020).

Regarding the role of teacher care on student grit, the SEM analysis also showed a significant path from teacher care to student grit. Students who feel cared for by their teachers are more likely to persevere through challenges and maintain their motivation over time. This relationship can be theoretically explained by Positive Psychology (PP) and Self-Determination Theory (SDT). PP highlights the importance of positive emotions and supportive relationships in fostering resilience and perseverance (MacIntyre et al., 2019). SDT emphasizes the role of supportive environments in promoting intrinsic motivation and self-determination, leading to higher levels of grit (Jeno et al., 2019).

The findings support the interconnected nature of teacher care, engagement, and grit. Teacher care acts as a catalyst, enhancing students’ emotional well-being and providing them with the psychological resources needed to engage actively in their learning and persist through difficulties. This interconnected influence is supported by the rhetorical/relational objective theory, which posits that teachers’ communicative behaviors fulfill both educational objectives and students’ psychosocial needs (Mottet et al., 2006). The conceptual frameworks underlying the present study, namely Positive Psychology (PP), Self-Determination Theory (SDT), and rhetorical/relational objective theory, support the findings. The impact of teacher care on the engagement of learners in striving for educational objectives in an EFL setting can be theoretically elucidated by considering the advancement of PP in language acquisition, employing a constructive approach for the growth of EFL educators and learners’ biological, individual, and interpersonal aspects (MacIntyre et al., 2019). Teachers’ care is essential in PP, which emphasizes the ways individuals can flourish and experience greater happiness. This approach highlights positive emotions such as optimism, passion, adaptability, positivity, and similar feelings, rather than destructive emotions (Derakhshan & Yin, 2024; Dewaele et al., 2019; Wang et al., 2021).

The findings maintain the rhetorical/relational objective theory and align with prior research that indicated elevated levels of teacher care resulted in enhanced performance, engagement, contentment, knowledge, well-being, and motivation among learners (Derakhshan, Doliński, et al., 2022; Derakhshan et al., 2019). Therefore, it appears that when educators demonstrate caring actions such as displaying empathy towards learners, sustaining visual contact with everyone, and exhibiting genuine curiosity in their academic progress, learners’ emotional and psychosocial requirements are fulfilled, making them more prone to attend classes consistently (Gabryś-Barker, 2016; Noddings, 2012). Based on this theory, the majority of educators’ communication actions have the potential to fulfill the objectives of educators and cater to the requirements of students (Myers et al., 2018). Consequently, it is contended that teacher care has been linked with several favorable results, such as enhanced attendance, heightened study duration, enhanced scholastic performance, reduced anxiety, and decreased attrition rate (Bieg et al., 2013).

As SDT focuses on how classroom settings affect individuals’ emotional requirements, teacher care aids in fostering the self-autonomy of learners and motivates them to investigate on their own, enabling them to actively confront learning obstacles (Jeno et al., 2019). Moreover, teacher care stimulates learners’ intrinsic motivation and increases their focus during the educational process, thereby resulting in increased engagement at a more advanced level. Based on SDT, educators are accountable for equipping learners with chances to embrace their principles and requirements and for offering captivating and contextually rich input and resources to learners (Zeng et al., 2019). In the same vein, the significance of constructive teacher relational factors for improving students’ achievement of positive educational results is demonstrated in the Xie and Derakhshan (2021) statement that educators can establish a pleasant learning atmosphere by demonstrating regard and care for learners, fostering constructive teacher-student relations, and cultivating curiosity in the target language. These actions will finally assist students in maintaining their engagement in L2 learning.

This outcome advocates the notion of Derakhshan et al. (2022) who posited that learners’ engagement in language courses could depend on the optimal connection between learner and instructor and the presence of considerable teacher care. Given that teacher affections have a significant impact on forming interpersonal connections with students in the classroom setting, their supportive actions can create an environment that promotes a positive bond with learners in the EFL class. As previously mentioned, teachers’ favorable interpersonal connections with the students can establish a trustworthy classroom environment that can ultimately enhance the probability of successful teaching and learning. In this kind of situation, students’ persistence and enthusiasm for language learning are more likely to be cultivated. The upshots are also in line with Eskreis-Winkler et al. (2014) who specified that support from educators was meaningfully related to grit. Moreover, when learners perceived increased teacher assistance, the connection between grit and academic success was more robust (Clark et al., 2020).

The findings of the current investigation also suggest that compassionate language instructors who willingly value their learners can make a caring environment in the educational setting, which bolsters the students’ grit to pursue their language learning goals over an extended period. The favorable impact of teacher nurturing on Chinese EFL learners' grit can be rationalized by the observation that learners who receive care and appreciation from their educators are more likely to persist in their learning endeavors despite encountering difficulties and obstacles (Lavy & Naama-Ghanayim, 2020). The results of this study serve as an indirect confirmation of Pan's (2022) research, which established a strong correlation between teachers' positive behaviors and students' levels of grit. The study found a positive correlation between teacher support and the level of grit displayed by Chinese learners. It is pertinent to contextualize this finding in comparison to the results reported by Shen and Guo (2022). It was found that there is a strong correlation between teacher support and students' persistence in their academic endeavors. This outcome also indirectly supports Pan's (2022) findings, which indicated that positive behaviors are strongly tied to student grit. The present finding also validates the results reported by Feng et al. (2019). Researchers established a significant correlation between the level of parental and teacher support and students' persistence and effort. This finding is further substantiated in consideration of social support theory, which posits that students' perception of their teachers as being nurturing and supportive provides them with an essential source of both emotional and instrumental support. This form of support serves as a protective factor against the adverse effects of stressors and ultimately promotes their motivation and involvement.

12.On page 10-11, “It is evident that when educators demonstrate care …performance and cognitive capability can be readily enhanced”. However, performance and cognitive capability are not mentioned at all in the model. Meanwhile, it is suggested that all the variables in the model should be explicitly listed and explained.

Response:

Thanks for the comments. This section was revised thoroughly as highlighted above.

13.It is suggested that subtitles be added in Part 5 and 6 to enhance readability.
Response:

Thanks for the comments. This is in line with the format of the journal. However, in order to consider the issue, they were broken into more paragraphs.

14.Language throughout the manuscript should be extensively revised so as to meet the requirements of an academic journal.

Response:

Thanks for the comments. Checked.

Reviewer #4: 1. The manuscript lacks a section dedicated to hypothesis development, where the authors should propose hypotheses based on a comprehensive literature review.

Response:

Thanks for the comments. Added.

H01: There is not any significant relationship between Chinese EFL students’ engagement, grit, and teacher care.

H02: Teacher care does not significantly predict student engagement and grit among Chinese EFL learners.

2. You suggest that student engagement consists of six elements: future goals, control of schoolwork, extrinsic motivation, family support, peer support, and teacher-student interactions. Why? It is necessary to discuss the rationale behind this composition, ideally grounded in one or more theoretical models.

Response:

Thanks for the comments. Added. This Scale is a comprehensive tool designed to assess various dimensions of student engagement. By evaluating emotional, behavioral, and cognitive aspects, this scale provides a holistic understanding of how students interact with their learning environment and what factors contribute to their overall engagement.

This scale, developed by Appleton et al. (2006), comprises 35 statements and utilizes a four-point Likert scale (fluctuating from strongly disagree to strongly agree). It aims to assess three distinct aspects of engagement: emotional, behavioral, and cognitive. Emotional engagement focuses on the bond between educators and learners, and the support received from peers and guardians. This aspect measures the quality of the relationship between students and their teachers and it looks at the encouragement, help, and emotional backing students get from their immediate social circles, which can influence their engagement in school. The other is behavioral engagement that measures participation in academic and extracurricular activities.This component evaluates students' involvement in academic tasks such as attending classes, completing assignments, and participating in discussions. Cognitive engagement assesses the quality of school assignments, goals and desires, and intrinsic motivation. Indeed, it examines students' internal drive to learn and succeed.The reliability of this questionnaire was found to be .879 through Composite Reliability (CR).

3. Does this framework genuinely based on the self-determined theory (SDT)? The self-determined theory (SDT) encompasses three elements: competence, autonomy, and relatedness. Are these three elements incorporated into your framework?

Response:

Thanks for the comments. More explanations were provided.

This research explored the power of teacher care in enhancing EFL learners’ engagement and grit in China. The findings from the SEM analysis demonstrated significant paths through which teacher care influences EFL learners’ engagement and grit. The SEM results indicated that teacher care directly predicts both engagement and grit among EFL learners, with about 59 percent of changes in grit and about 67 percent of changes in engagement being explained by teacher care. This robust correlation underscores the critical role of teacher care in fostering a supportive learning environment that enhances both emotional and behavioral student outcomes.

Regarding the role of teacher care on student engagement, the path analysis revealed that teacher care significantly enhances student engagement. When students perceive their teachers as caring, it provides them with emotional and instrumental support, which buffers against stress and promotes their involvement in academic activities. This finding is also justified in light of social support theory which suggests that when students perceive their teachers as caring and supportive, they have access to a valuable source of emotional and instrumental support. This support buffers the negative impact of stressors and ultimately contributes to their engagement (Havik & Westergård, 2020).

Teacher care acts as a catalyst, enhancing students’ emotional well-being and providing them with the psychological resources needed to engage actively in their learning and persist through difficulties. This interconnected influence is supported by the rhetorical/relational objective theory, which posits that teachers’ communicative behaviors fulfill both educational objectives and students’ psychosocial needs (Mottet et al., 2006). The impact of teacher care on the engagement of learners in striving for educational objectives in an EFL setting can be theoretically elucidated by considering the advancement of PP in language acquisition, employing a constructive approach for the growth of EFL educators and learners’ biological, individual, and interpersonal aspects (MacIntyre et al., 2019). Teacher care is essential in PP, which emphasizes the ways individuals can flourish and experience greater happiness. This approach highlights positive emotions such as optimism, passion, adaptability, positivity, and similar feelings, rather than destructive emotions (Derakhshan & Yin, 2024; Dewaele et al., 2019; Wang et al., 2021).

The findings maintain the rhetorical/relational objective theory and align with prior research that indicated elevated levels of teacher care resulted in enhanced performance, engagement, contentment, knowledge, well-being, and motivation among learners (Derakhshan, Doliński, et al., 2022; Derakhshan et al., 2019). Therefore, it appears that when educators demonstrate caring actions such as displaying empathy towards learners, sustaining visual contact with everyone, and exhibiting genuine curiosity in their academic progress, learners’ emotional and psychosocial requirements are fulfilled, making them more prone to attend classes consistently (Gabryś-Barker, 2016; Noddings, 2012). Based on this theory, the majority of educators’ communication actions have the potential to fulfill the objectives of educators and cater to the requirements of students (Myers et al., 2018). Consequently, it is contended that teacher care has been linked with several favorable results, such as enhanced attendance, heightened study duration, enhanced scholastic performance, reduced anxiety, and decreased attrition rate (Bieg et al., 2013).

In line with SDT, teacher care stimulates learners’ intrinsic motivation and increases their focus during the educational process, thereby resulting in increased engagement at a more advanced level. Based on SDT, educators are accountable for equipping learners with chances to embrace their principles and requirements and for offering captivating and contextually rich input and resources to learners (Zeng et al., 2019). As SDT focuses on how classroom settings affect individuals’ emotional requirements, teacher care aids in fostering the self-autonomy of learners and motivates them to investigate on their own, enabling them to actively confront learning obstacles (Jeno et al., 2019). In the same vein, the significance of constructive teacher relational factors for improving students’ achievement of positive educational results is demonstrated in the Xie and Derakhshan (2021) statement that educators can establish a pleasant learning atmosphere by demonstrating regard and care for learners, fostering constructive teacher-student relations, and cultivating curiosity in the target language. These actions will finally assist students in maintaining their engagement in L2 learning. This outcome advocates the notion of Derakhshan et al. (2022) who posited that learners’ engagement in language courses could depend on the optimal connection between learner and instructor and the presence of considerable teacher care. Given that teacher affections have a significant impact on forming interpersonal connections with students in the classroom setting, their supportive actions can create an environment that promotes a positive bond with learners in the EFL class.

Regarding the role of teacher care on student grit, the SEM analysis also showed a significant path from teacher care to student grit. Students who feel cared for by their teachers are more likely to persevere through challenges and maintain their motivation over time. This relationship can be theoretically explained by PP and SDT. PP highlights the importance of positive emotions and supportive relationships in fostering resilience and perseverance (MacIntyre et al., 2019). SDT emphasizes the role of supportive environments in promoting intrinsic motivation and self-determination, leading to higher levels of grit (Jeno et al., 2019).

In line with SDT, teacher care addresses three psychological needs, thereby increasing their level of grit. For instance, competence is cultivated when teachers provide positive feedback and set stimulating goals and when students believe they can deal with setbacks, they are more prone to persevere in their efforts, a key aspect of grit (Jeno et al., 2019). Indeed, the feedback and guidance provided by caring teachers help students develop resilience and a long-term commitment to their goals.Moreover, teacher care can inspire self-directed learning as it raises a sense of ownership and accountability towards one’s learning, which drives long-standing commitment and perseverance (Zeng et al., 2019). Also, through the supportive relationships, students feel understood, valued, and connected to their teachers, they preserve their effort and interest over prolonged periods, even in the case of challenges (Xie & Derakhshan, 2021). Indeed, teachers’ favorable interpersonal connections with the students can establish a trustworthy classroom environment that can ultimately enhance the probability of successful teaching and learning. In this kind of situation, students’ persistence and enthusiasm for language learning are more likely to be cultivated. The favorable impact of teacher care on Chinese EFL learners' grit can be rationalized by the observation that learners who receive care and appreciation from their educators are more likely to persist in their learning endeavors despite encountering difficulties and obstacles (Lavy & Naama-Ghanayim, 2020). The upshots are also in line with Eskreis-Winkler et al. (2014) who specified that support from educators was meaningfully related to grit. Moreover, when learners perceived increased teacher assistance, the connection between grit and academic success was more robust (Clark et al., 2020). The results of this study serve as an indirect confirmation of Pan's (2022) research, which established a strong correlation between teachers' positive behaviors and students' levels of grit. It is pertinent to contextualize this finding in comparison to the results reported by Shen and Guo (2022). The present finding also validates the results reported by Feng et al. (2019) as they established a significant correlation between the level of parental and teacher support and students' persistence and effort.

4. If extrinsic motivation can enhance student engagement, what about intrinsic motivation? Why is intrinsic motivation omitted from the framework?

Response:

Thanks for the comments. Based on the engagement questionnaire, both concepts has been taken into consideration. Indeed, intrinsic motivation was measured through goals and desires, the aspects of cognitive engagement in this scale. More explanations were provided in discussion.

5. The self-determined theory (SDT) was not introduced by Ryan and Deci in 2020; it was proposed approximately four decades ago. Although these authors did publish a related article in 2020, it is imperative for academic manuscripts to cite the foundational literature when referencing significant theories.

Response:

Thanks for the comments. yes exactly. Indeed, the foundational concepts of SDT were introduced by Deci and Ryan in the 1980s (Deci & Ryan, 1985). However, in 2020, Ryan and Deci delved deeper into the nuances of how autonomy, competence, and relatedness contribute to motivation and psychological well-being. Added.

SDT suggests that the care from others, such as parents and teachers, is essential for learners’ achievement that this care helps satisfy the three basic psychological needs: competence, autonomy, and relatedness (Deci & Ryan, 1985).

6. There is a logical gap in the manuscript. In section 2.3, you reference the self-determined theory (SDT) without explaining what it is or how it relates to teacher care (the focus of that section). While you mention "support from others," there is a logical gap between teacher support and teacher care that the authors need to address.

Response:

Thanks for the comments. More explanations were provided. No the focus is on teacher care that was revised.

SDT suggests that the care from others, such as parents and teachers, is essential for learners’ achievement that this care helps satisfy the three basic psychological needs: competence, autonomy, and relatedness (Deci & Ryan, 1985). SDT is an instructional theory of motivation that is based on the important value of learner autonomy (Ryan & Deci, 2020). The SDT suggested that care of others such as parents and instructors is essential for students’ success (Ryan & Deci, 2020).

7. Section 2.4 is not justified. A literature review typically involves examining related studies, making the inclusion of a separate subsection for "related studies" unnecessary and odd.

Response:

Thanks for the comments. These studies collectively highlight the critical role of teacher care in various aspects of student achievement, well-being, and engagement, underscoring the importance of nurturing teacher care to promote positive educational outcomes.

8. There is an issue with the formulation of the first research question. Please reword the first research question.

Response:

Thanks for the comments.. However, the original formulation of the first research question is clear and aligns well with the study's objectives. It directly addresses the investigation of the relationship between Chinese EFL students’ engagement, grit, and teacher care.

9. The data analysis subsection within the "Methods" section is inadequate. Please provide more specificity in the data analysis process. The criterion is whether readers, without looking at the findings section, can understand the steps of your data analysis from the methods section alone.
Response:

Thanks for the comments.More explanations were provided.

The data were analyzed using Structural Equation Modeling (SEM) to examine the relationships between teacher care, student engagement, and grit. Descriptive statistics, including means, standard deviations, and frequency distributions, were first calculated to provide an overview of the participants' responses. The internal consistency of each instrument was confirmed through Composite Reliability (CR). SEM was then employed to test the hypothesized model and assess the direct and indirect effects of teacher care on engagement and grit. Linear Regression analysis was also conducted to further explore the predictor roles of variables.

10. It is recommended that the Conclusion section be organized in the "summary-implication-limitations-future work" structure.

Response:

Thanks for the comments. Reorganized.

It is presumed that when there is good interaction between learners and educators, students can undergo positive emotions which are essential to successful teaching and learning (Seligman & Csikszentmihalyi, 2014). Teacher care as a positive instructor communication can be elucidated in the context of PP which has garnered significant attention over the past two decades, encompassing three primary pillars; namely constructive encounters, constructive individual attributes, and constructive organizations (Seligman, 2018). Consistent with the results of a prior investigation conducted by Lavy and Naama-Ghanayim (2020), the outcomes of the current study proved that there is a simultaneous occurrence of elevated perceptions of care and engagement. This can be academically illuminated by the interconnectedness of language education, which compels language teachers to utilize affirmative (non)verbal signals of communication and convey positive feelings and emotions to students within the language learning setting, thereby establishing a socially and interpersonally conducive learning context (Mercer & Gkonou, 2020). Likewise, as suggested by the advocates of PP in EFL education (Wang et al., 2022), since positive feelings of educators and relational actions are powerful predictors of any positive EFL learning concepts, they also foster learners’ engagement (Hiver et al., 2021). When learners are instructed by teachers who are concerned about them, they experience positive educational results such as increased resourcefulness, greater contentment, and perseverance. These positive responses and enthusiasm are essential for their active engagement in the learning process and contribute to their grit. This is because when teachers identify and nurture students’ aspirations, inclinations, and interests, they create supportive classroom environments that promote learning (Hang et al., 2017).

Teacher trainers are accountable for equipping L2 instructors who, among other duties, possess strong interpersonal skills in establishing a welcoming and friendly classroom environment, engaging in effective communication with learners, and demonstrating culturally sensitive care towards them (Mercer & Gkonou, 2020). Managers of language schools and committees responsible for recruiting language teachers can expand the range of criteria for selecting effective teachers in a manner that includes not only linguistic expertise, but also a strong comprehension of psychological, interpersonal, emotional, and cultural elements of language instruction. These elements are significant in defining the efficacy of the knowledge that EFL teachers impart to their learners (Pishghadam et al., 2021).

The present study is subject to certain limitations, and it is advisable to exercise caution when generalizing the findings. The inclusion of self-reported data in testing the model necessitates a mixed-methods approach to obtain more comprehensive insights, ultimately enhancing our understanding of the relationships between key variables. Furthermore, it is important to note that all participants in this study were EFL students. This raises the need for further research to be conducted specifically with ESL teachers to validate the proposed model within this particular context. This study had some other constraints that must be considered when describing the results obtained. For example, this study employed a quantitative approach; thus, additional inquiries utilizing a combination of methods are required to provide a more holistic view of the association between teacher care, grit, and engagement. In addition, this research did not investigate the demographic factors of college educators or students and their possible correlations with the study's variables; hence, these factors could present an interesting line for future investigation. It would be noteworthy to assess the moderating effects of issues such as age, level of education, and area of specialization on the relationships between the concepts. Additional research will investigate the enabling or regulating observable actions of more resilient students that will yield academic advancements of resilience and contribute to the development of grit-improving treatment programs. It is advisable to conduct a longitudinal form of investigation because, firstly, time plays a crucial role in acquiring more evident and advantageous information from the subjects (Saito et al., 2018), and secondly, the grit scale intends to assess an individual's ambition and persistence towards enduring goals.

11. The Introduction section should follow the structure of "background-gaps-aims and significance-research questions." In the current manuscript, the research gaps are not clearly highlighted.

Response:

Thanks for the comments. Added.

While various elements are essential for effective language instruction, psycho-emotional traits appear to have a greater impact on learning and were largely disregarded in constructivism (Sikma, 2021). As a recent development in the field of psychology, known as Positive Psychology (PP), significant focus has been placed on the strength and impact of constructive feelings in achieving a more joyful existence and a successful profession (Gregersen & MacIntyre, 2021; Wang et al., 2021). As per the principles of PP, grit, and engagement have a substantial influence on L2 education as they strive to improve educational achievements and cultivate conducive learning environments (Chen & Shu, 2024; Derakhshan, Greenier, et al., 2022; Liu & Wang, 2021). Learner success was significantly impacted by grit, as a psychological characteristic (Datu et al., 2016; Derakhshan, Dewaele, et al., 2022; Wang et al., 2021). Grit, as a relatively novel concept in the PP framework, includes the theories of passion and perseverance, which have received interest in diverse domains, including professional and educational settings (MacIntyre et al., 2019). Also, it is said to be a substantial factor in the language instruction procedure and it is recognized in the collection of works as a valuable subject for individuals who are prone to flourish in education as it has been intertwined with enhanced dedication (Derakhshan, Solhi, et al., 2023; Hodge et al., 2018). Now, educators and professionals are expected to motivate and cultivate students’ abilities and persistence to work vigorously to accomplish their ultimate objectives (Taspinar & Kulekci, 2018). Furthermore, Pawlak et al. (2022) proposed that gritty individuals are motivated by a personal fascination in the route of acquiring knowledge, and those with individual objectives experience a heightened level of motivation, confidence, and enthusiasm, leading to enhanced achievement, grit, and well-being. While grit has been comprehensively inspected in educational psychology, it has recently been taken into the scrutiny of EFL researchers (Pawlak et al., 2022; Yang et al., 2022).

Further, as PP gained attention in the field of educational psychology, alongside grit, educators comprehended the importance of giving greater attention to the engagement of learners as a crucial element in fostering academic achievement (Pishghadam et al., 2019; Zhang, 2021). The engagement of students has been extensively studied in various fields (Oga-Baldwin, 2019). Since learners’ engagement has a pivotal part in enhancing their learning success motivating the learners to actively participate in the learning route has always been a primacy for educators in every educational domain and it is deemed essential for academic achievement, persistence, retention, and academic success (Gunuc & Kuzu, 2015). Given that engagement in the pursuit of educational objectives in a language learning setting is of utmost prominence for the academic achievement of EFL learners, it is considered essential to elucidate the favorable factors that foster learner engagement within the realm of EFL investigation (Khajavy, 2021). The effectiveness of language acquisition in the educational setting is contingent upon the instructor, and it is asserted that the learner’s progress in learning may hinge on the competence of the educators (Pishghadam et al., 2019). In self-determination theory (SDT), learners’ engagement might be greatly improved through their connection with their instructor (Fredricks et al., 2016). Given the regular exchanges between learners and instructors in language classes, instructors are seen as a crucial means of assistance for learners during classroom activities and learning procedures (Ryan & Deci, 2020). In the same vein, Duckworth (2016) suggested that educators can be considered as the significant factors for the enhancement and advancement of grit. Also, learners’ engagement can be improved through a thoughtful combination with other favorable concepts such as encouragement, connection, social assistance, and care. Teachers can assume diverse functions like being mindful of feedback to learners, offering questions to engage them, and reevaluating classroom administration to regulate relations (Mercer & Gkonou, 2020).

More specifically, teacher care refers to teachers’ spoken and non-spoken efforts to fulfill the emotional and mental requirements of students (Noddings, 2012). Likewise, caring has been described as the emotions, actions, and ideas that arise from an educator’s wish to inspire, assist, involve, or motivate their students (O’Connor, 2008). In the realm of academics, the notion of ‘care’ has been extensively studied and is developing as a crucial factor in successful education (Pishghadam et al., 2019). Teacher care has a substantial role in improving learners’ engagement and success and fostering the social and ethical growth of learners (Lam et al., 2012). Educators can create a caring atmosphere in which communications are more meaningful when compared to other tactics (Pappano, 2013). Focusing on the reciprocal aspect of this concept, it is argued that ‘caring’ is the action that emerges from a mutual caring connection between student and instructor, where knowledge acquisition occurs by modeling, conversation, and endorsement at the societal levels (Velasquez et al., 2013). Teacher care motivates student-associated skills such as engagement, mental health, and accomplishment (Derakhshan et al., 2019; Havik & Westergård, 2020).

Although teacher care has been explored in terms of engagement in general instruction (e.g., Dickinson & Kreitmair, 2019; Lewis et al., 2012; Lavy & Naama-Ghanayim, 2020), it remains an area that lacks extensive investigations in the field of language learning. For example, Pishghadam et al. (2019) and Yuan (2022) conducted a limited number of inquiries that concentrated on the impact of teacher stroking behavior, which is a key aspect of teacher care, on learners’ success in the EFL context. Teacher care not only encompasses verbal and non-verbal communication strategies—such as stroking behavior—but also includes emotional support, empathy, and responsiveness to students' needs. These elements are crucial for creating a sense of belonging and safety, which are essential for optimal learning. Based on the literature, when learners perceive their teachers as caring, they are more prone to involve intensely in the path of learning, show more motivation, and cultivate resilience in the case of difficulties (Khajavy, 2021). This implies that teacher care could act as a key element that impacts academic success.

However, there is still a lack of understanding regarding the influence of teacher care on both grit and engagement of learners in the context of EFL students, especially in China. When considering the combined significance of teacher-caring behavior as a form of positive interpersonal communication in language education, alongside learner engagement and grit, it becomes necessary to prioritize the investigation of the association between these three constructs. Moreover, there has been limited research on the correlation between student engagement, grit, and teacher-caring behavior within the realm of language education. This study aims to examine the specific impact of teacher care on students' grit and involvement, to inform strategic interventions and methodologies that promote increased grit and improved engagement in EFL learning environments. By addressing the identified gaps, this research provides significant insights for educators and policymakers, which can aid in the development of more supportive and enriching learning environments for EFL learners. Finally, this can lead to an increase in student grit and engagement.

12. The final paragraph of the Introduction is excessively long; it is advised to break it into smaller sections.

Response:

Thanks for the comments. Done.

13. Sections 2.1 and 2.2 each contain a single paragraph that is excessively long. It is recommended that these paragraphs be broken down into shorter, more manageable sections.

Response:

Thanks for the comments. Done.

14. Please provide the full name when any abbreviation is used for the first time, such as SDT.

Response:

Thanks for the comments. yes we did.

15. The capitalization of keywords is inconsistent.

Response:

Thanks for the comments. Revised .
