# Supplementary material for: The Relationship between students’ engagement, grit, and teacher care in Chinese context
Source: PLoS One. 2025 Jul 3;20(7):e0326485. doi: 10.1371/journal.pone.0326485 (PMC12225857; doi:10.1371/journal.pone.0326485)
Supplement: Attachment — Submitted filename: Response.docx [file pone.0326485.s003.docx]

Dear Editors and Reviewers,

We are delighted to hear that the venerable reviewers found our study interesting. We, the authors, hereby do appreciate the insightful and constructive comments, which have absolutely added to the rigor and robustness of the revised manuscript.

We did our utmost to attend to all precious comments as meticulously as we could to fulfil the esteemed reviewer’s expectations in this round. We will also apply any other modifications suggested in the next revision.

**Additional Editor Comments:**

Thank reviewers for their valuable feedback. Additionally, please pay attention to the following points:
1. Table Sequence: In the manuscript, Table 3 should be followed by Table 4, but it is currently followed by Table 1. Please revise.

**Response**

Thank you for the comment. The sequence of the tables were checked out and revised.

2. Table Headers: Each table should include clear and descriptive headers. For example, a header such as "gender" clarifies that the entries should be "male" or "female." Currently, the second column of Table 1 and the first column of Table 2 lack headers. Please ensure all columns are appropriately labeled.
**Response**

Thank you for the comment. All columns were labeled.

3. RMSEA Benchmark: In Table 1, the RMSEA benchmark is cited as 0.06. However, based on my review of the literature, the accepted benchmark is 0.05. Please either provide a credible reference supporting the 0.06 threshold or revise the value to 0.05 after verification.
**Response**

Thank you for the comment. For RMESA, we followed Hu and Bentelr’s (1999) benchmarks and the reference is added to the manuscript.

The thresholds in Table 1 are the ones proposed by Hu and Bentler (1999), according to which the obtained model fit indices are considered satisfactory to excellent.

4. Model Fit Interpretation: The interpretation of model fit indices requires revision. For instance, a CMIN/df value between 1 and 3 is typically considered excellent, rather than the current interpretation provided. Please carefully review and update the criteria used for evaluating model fit.
**Response**

Thank you for the comment. We revised the interpretation in the table.

Table 1

*Assessment of the CFA Goodness of Fit*

|  |  |  | Threshold |  |  |
| --- | --- | --- | --- | --- | --- |
| Criteria |  | Terrible | Satisfactory | Excellent | Evaluation |
| CMIN | 5720.793 |  |  |  |  |
| DF | 2130 |  |  |  |  |
| CMIN/DF | 2.686 | > 5 | > 3 | > 1 | Excellent |
| RMSEA | .065 | > 0.08 | < 0.08 | < 0.06 | Satisfactory |
| GFI | .932 | < 0.9 | > 0.9 | > 0.95 | Satisfactory |
| CFI | .930 | < 0.9 | > 0.9 | > 0.95 | Satisfactory |
| PNFI | .609 | < 0.5 | > 0.5 |  | Excellent |
| TLI | .920 | > 0.9 | > 0.9 | > 0.95 | Satisfactory |

5. Instrument presentation. While the sources of each scale are clearly stated, enhancing transparency and reproducibility would be beneficial. Consider adding a table listing all survey items used in the study. If this table is included, please ensure each item is appropriate and accurately represents the constructs measured. This is a suggestion for improvement (not mandatory).

**Response**

Thank you for the comment. The necessary information and the components have been already mentioned.

6. Long paragraphs. Some paragraphs in the introduction are excessively long, spanning half a page or more. To improve readability and logical flow, consider breaking these into shorter, more focused paragraphs. This will help emphasize key points and enhance the overall coherence of the section. This is a suggestion for improvement (not mandatory).

**Response**

Thank you for the comment. Done.

7. Visual presentation: The current presentation of Figures 1 and 2 does not meet standard aesthetic guidelines. For consistency and clarity:
- Ensure graphical elements representing concepts at the same level are of uniform size.
- Align items vertically unless there is a specific rationale for deviation.
- Distribute lines and elements evenly to avoid visual imbalance.
For example, in Figure 1, the element "student engagement" connects to "future goals" and "teacher-student interactions," but the boxes for these elements are inconsistently sized, misaligned, and unevenly spaced relative to the central element. Please revise these figures to adhere to best practices in visual presentation.
**Response**

Thank you for the comment. Figure 1 was thoroughly revised. Figure 2 was deleted as the figure does not add new information.

Figure 1. *The Final Measurement Model* *with Standardized Estimates*

**Reviewer #1**

The revised manuscript shows significant improvements, particularly in the methodology and discussion sections, and the major concerns have been appropriately resolved. However, I recommend a thorough proofreading to enhance linguistic and logic accuracy. For example:

1. The title of Table 3 contains a punctuation mistake: The Results of "Path Analysis with SEM.
**Response**

Thank you for the comment. We corrected the mistake.

Table 3

*The Results of Path Analysis with SEM*

|  |  |  | Estimate | S.E. | C.R. | P |
| --- | --- | --- | --- | --- | --- | --- |
| Grit | <--- | Teacher Care | .593 | .040 | 9.079 | .000 |
| Student Engagement | <--- | Teacher Care | .674 | .031 | 7.976 | .000 |

2. In the discussion of CMIN/DF (2.686), it should be described as "excellent" rather than "satisfactory", in accordance with the standard model fit criteria you present in Table 1. Because the standard in your Table shows that ">3 is satisfactory, >1 is excellent".

**Response:**

Thank you for the comment. We revised the interpretation in the table.

Table 1

*Assessment of the CFA Goodness of Fit*

|  |  |  | Threshold |  |  |
| --- | --- | --- | --- | --- | --- |
| Criteria |  | Terrible | Satisfactory | Excellent | Evaluation |
| CMIN | 5720.793 |  |  |  |  |
| DF | 2130 |  |  |  |  |
| CMIN/DF | 2.686 | > 5 | > 3 | > 1 | Excellent |
| RMSEA | .065 | > 0.08 | < 0.08 | < 0.06 | Satisfactory |
| GFI | .932 | < 0.9 | > 0.9 | > 0.95 | Satisfactory |
| CFI | .930 | < 0.9 | > 0.9 | > 0.95 | Satisfactory |
| PNFI | .609 | < 0.5 | > 0.5 |  | Excellent |
| TLI | .920 | > 0.9 | > 0.9 | > 0.95 | Satisfactory |

**Reviewer #3**

1. The whole section of “Literature Review” should be the review of related studies. In this case, the subtitle of 2.4 “Related Studies” is improper.

**Response**

Thank you for the comment. The title was changed to **Empirical Studies.**

2. In this study, two research questions and two research hypotheses were proposed. From RQ1, it appears that the authors aim to explore the relationship among “Chinese EFL students’ engagement, grit, and teacher care”, while H01 states that there is no relationship among them. This is quite odd and perplexing. The premise of RQ2 is that teacher care can predict student engagement and grit among Chinese EFL learners, but it is hypothesized in H02 “Teacher care does not significantly predict student engagement and grit among Chinese EFL learners”.
Moreover, in the results and discussion sections, the authors primarily focus on addressing the research questions, with nothing concerning the research hypotheses. In fact, as the authors stated the research indeed affirms that “teacher care plays a significant role in influencing students' engagement and grit”, which rebuts H01. Therefore, it is strongly recommended that the authors re-consider the logical consistency between RQs and the necessity of proposing the hypotheses.

**Response**

Thank you for the comment. To resolve this issue, we have revised our hypotheses to better reflect the intent of our research questions and align them with our analytical approach:

**H01**: There is a significant relationship between Chinese EFL students’ engagement, grit, and teacher care.

**H02**: Teacher care significantly predicts student engagement and grit among Chinese EFL learners.

3. Please modify the table number. There are two tables numbered “Table 1”, the second of which should be “Table 4”.
**Response**

Thank you for the comment. We revised the sequence number of the tables.

4. There exist inaccuracy and inconsistency in some sentences like “The results of Table 3 signify that about 59 percent of variations in the EFL students’ grit can be predicted by their teacher care, and about 67 percent of variations in the EFL student engagement can be predicted by their teachers’ care.” Please check the language to ensure consistency and accuracy.

**Response**

Thank you for the comment. The statement was revised.

*Table 4*

*Structural Model Assessment*

| PARAMETER | ESTIMATE | LOWER | UPPER | P |
| --- | --- | --- | --- | --- |
| Student engagement | .453 | .159 | .356 | .001 |
| Grit | .354 | .256 | .578 | .001 |

Table 4 presents the results of a structural model assessment, focusing on the relationships between two parameters, student engagement and grit. The structural model assessment highlights significant positive contributions of both student engagement (Estimate = .453, CI [.159, .356], *p* = .001) and grit (Estimate = .354, CI [.256, .578], *p* = .001) to the model. These results underscore the critical roles of engagement and grit in the underlying structure of the observed phenomena. The results reveal that teacher care plays a significant role in influencing students' engagement and grit, highlighting its importance in the educational context. Specifically, approximately 45% of the variations observed in students' engagement can be directly attributed to the effect of teacher care. This indicates that nearly half of the students' motivation, participation, and emotional investment in learning activities are shaped by how much they perceive their teachers as supportive and empathetic. Similarly, about 35% of the variations in students' grit, which encompasses perseverance and passion for long-term goals, are also linked to the influence of teacher care. This suggests that a caring teacher-student relationship not only fosters immediate academic engagement but also cultivates essential character traits that drive sustained effort and resilience. These findings emphasize the transformative potential of teacher care in enhancing both the emotional and behavioral dimensions of students' academic experiences. Moreover, the results align with existing research, which underscores the critical role of supportive teaching practices in fostering positive student outcomes, thereby offering valuable insights for educators and policymakers aiming to optimize learning environments.
